# Cloning, expression and purification of Chikungunya virus E2 recombinant protein in *E.coli*

**DOI:** 10.1186/1471-2334-14-S3-P65

**Published:** 2014-05-27

**Authors:** Anil Kr Verma, Anmol Chandele, Murali-Krishna Kaja, Arockiasamy Arulandu, Pratima Ray

**Affiliations:** 1 Department of Pediatrics, All India Institute of Medical Sciences, Ansari Nagar, New Delhi, India

## Background

Chikungunya virus infection is an emerging arbovirus disease. While many infected individuals recover after primary illness, others suffer from persistent debilitating arthralgia that can last for months to years. Currently, there are no immune correlates, vaccines, therapies or robust diagnostics that can affectively prevent, treat or diagnose Chikungunya. Therefore, this study focuses on development of a better diagnostic assay for Chikungunya infection.

## Methods

We have initiated this study by cloning a functional form of Chikungunya virus E2 envelope protein. Sequence specific primers were designed based on the Chikungunya virus (African strain) E2 mRNA sequence obtained from NCBI (AF369024). This 846 base pair fragment was cloned in pET28b expression vector that added a His tag at the N terminus. E2 protein that was expressed in *E.coli* BL21 (DE3) strain and purified with Ni nTA affinity chromatography. The purified rHis e2 protein was characterized by SDS - PAGE and western blotting using an anti-His monoclonal antibody.

## Results

The E2 protein of the Chikv virus was successfully cloned and characterized as a 37KD protein. This purified protein worked at concentration of 2.5 µg/mL and detected both IgG and IgM antibodies from the plasma of Chikungunya patients and did not show non specific reactivity to normal (healthy) control plasma.

## Conclusion

We have successfully cloned the ChikvE2 protein and we are currently in the process of evaluating its use and efficiency in functional assays such as B cell ELISPOT assay and ELISA to be used for confirmatory diagnosis of Chikungunya.

